# Supplementary material for: Direct visualization of translesion DNA synthesis polymerase IV at the replisome
Source: Proc Natl Acad Sci U S A. 2022 Sep 19;119(39):e2208390119. doi: 10.1073/pnas.2208390119 (PMC9522359; doi:10.1073/pnas.2208390119)
Supplement: Supplementary File [file pnas.2208390119.sapp.pdf]

PNAS

Supporting Information for

Direct Visualization of Translesion DNA Synthesis Polymerase IV at the Replisome

Pham Minh Tuan[1], Neville S. Gilhooly[1,2], Kenneth J. Marians[3]*, and Stephen C. Kowalczykowski[1]*

[1]Department of Microbiology and Molecular Genetics and Department of Molecular and Cellular Biology, University of California, Davis, Davis, CA 95616; [3]Molecular Biology Program, Memorial Sloan Kettering Cancer Center, New York, NY 10065

[2]Current Address: Institute of Cancer and Genomic Sciences, Birmingham Centre for Genome Biology, College of Medical and Dental Sciences, University of Birmingham, Vincent Drive, B15 2TT, Birmingham UK

*Correspondence; K.J.M.: kmarians@sloankettering.edu, Tel. (212) 639-5890; S.C.K.: sckowalczykowski@ucdavis.edu, Tel. (530) 752-5938

This PDF file includes:

Figures S1 to S3
Legends for Movies S1 to S3

Other supporting materials for this manuscript include the following:

Movies S1 to S3

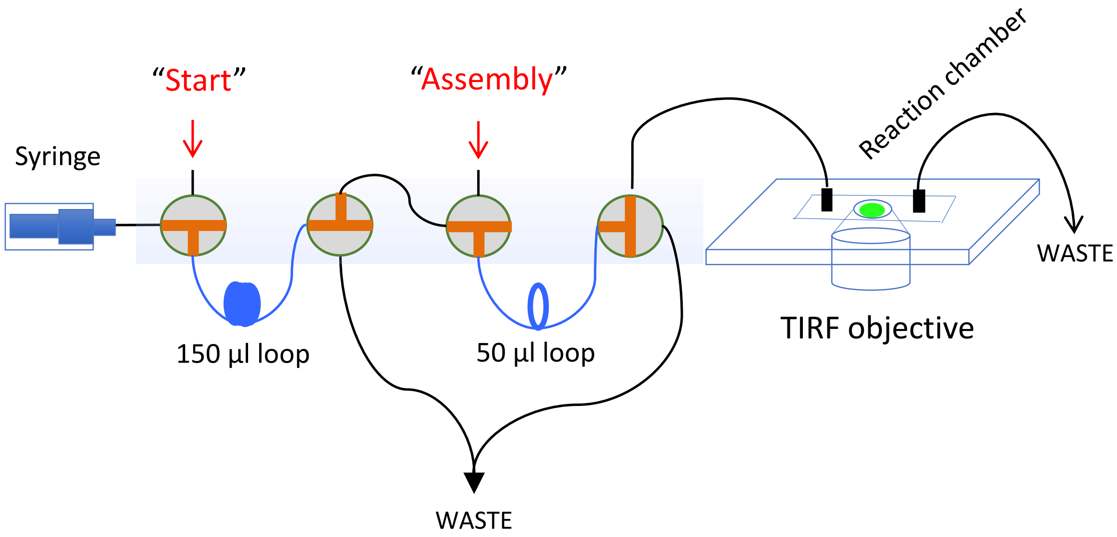

Fig. S1. Schematic representation of TIRF single molecule assay, related to Figure 1. The Assembly and Start reactions are loaded into 50 µl and 150 µl loops, respectively. The pump pushes the reactions to the chamber at a flow rate 1250 µl/hr.

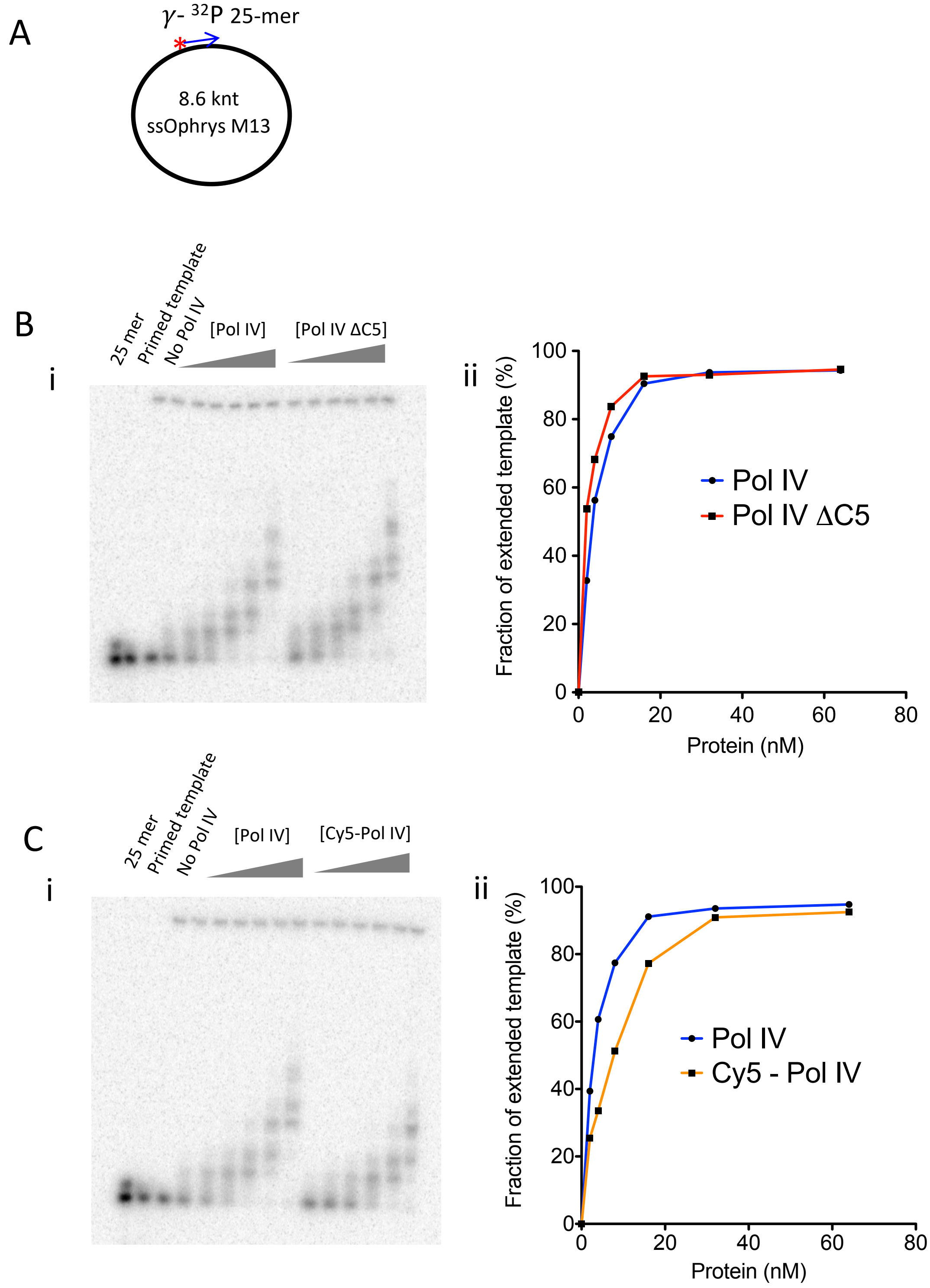

Fig. S2. Activity of Pol IV, Pol IV ΔC5 and Cy5-Pol IV, related to Figures 3 and 4. (A) Primer/Ophrys M13 DNA template. A ^{32}P–labeled 25-mer primer annealed to 8.6 knt Ophrys M13 circular ssDNA. (B) Extended products in presence of Pol IV and Pol IV ΔC5 (i) at 0, 2, 4, 8, 16, 32, and 64 nM; (ii) Graph showing fraction of extended products. (C) Extended products in presence of Pol IV and Cy5-Pol IV (i) at 0, 2, 4, 8, 16, 32, and 64 nM; (ii) Graph showing fraction of extended products.

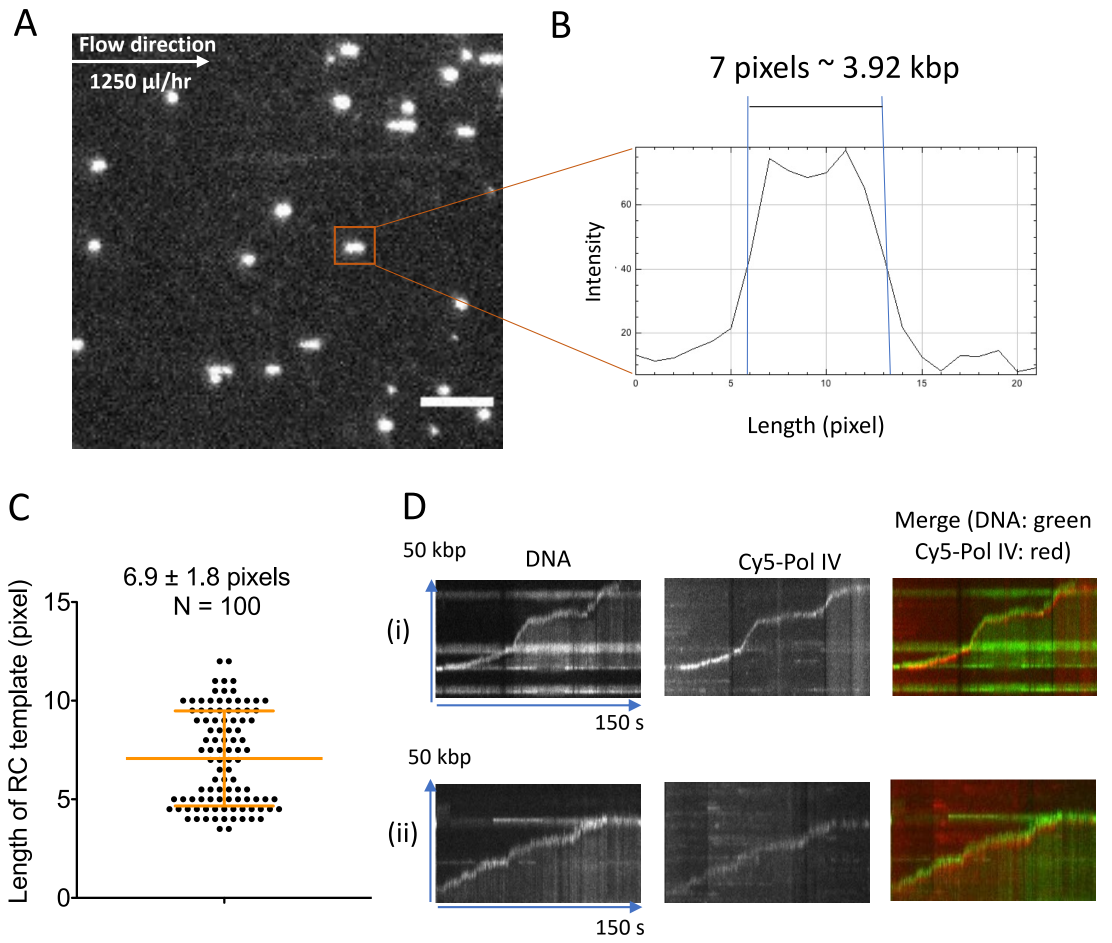

Fig. S3. Determining the distance from center of RC DNA template to the replisome, related to Figure 3. (A) Typical image of RC DNA template stained by SYTOX Orange on the glass surface under flow before loading the Assembly and Start reactions. Scale bar is 5 µm. (B) The length of RC DNA template is determined by using intensity profile of the molecules. (C) Average length of RC template under the flow. (D) (i) (ii) Kymographs of two typical molecules from two independent reactions showing live imaging of Cy5-Pol IV.

Movie S1 (separate files). Direct visualization of replication by live imaging in the absence of Pol IV, related to Figure 1. A) Movie of the same field as Figure 1B. The red “x” marks the molecule analyzed in Figure 1C. B) Movie of the same molecule as Figure 1C. Scale bar, 5 μm, equivalent to 17.5 kb dsDNA.

Movie S2 (separate files). Direct visualization of replication by live imaging in presence of Pol IV in the Assembly reaction, related to Figure 2. A) Movie of a field for a standard Pol III HE reaction containing 200 nM Pol IV. The red “x” marks the molecule analyzed in Figure 2A and B, iv. B) Movies (i), (ii), (iii), (iv), and (v) are of the molecules that were analyzed in Figures 2A and 2B, panels (i), (ii), (iii), (iv), and (v), respectively. Scale bar, 5 μm, equivalent to 17.5 kb dsDNA.

Movie S3 (separate file). Direct visualization of Cy5-Pol IV at the replisome by live imaging, related to Figure 3. Composite image is made by overlaying the two color channels (DNA is green and Cy5-Pol IV is red). Scale bar, 5 μm, equivalent to 17.5 kb dsDNA.
